# A dataset of distribution and diversity of mosquito-associated viruses and their mosquito vectors in China

**DOI:** 10.1038/s41597-020-00687-9

**Published:** 2020-10-13

**Authors:** Evans Atoni, Lu Zhao, Cheng Hu, Nanjie Ren, Xiaoyu Wang, Mengying Liang, Caroline Mwaliko, Zhiming Yuan, Han Xia

**Affiliations:** 1 Key Laboratory of Special Pathogens and Biosafety, Wuhan Institute of Virology, Chinese Academy of Sciences, Wuhan, Hubei China; 2 University of Chinese Academy of Sciences, Beijing, China; 3 BeiDou SuperSIT Spatial Information Industry (Wuhan) Inc, Wuhan, China

**Keywords:** Virology, Microbiology

## Abstract

Mosquito-borne viruses such as Zika virus, Japanese Encephalitis virus and Dengue virus present an increasing global health concern. However, in-depth knowledge of the distribution and diversity of mosquito-associated viruses and their related vectors remains limited, especially for China. To promote their understanding, we present the first comprehensive dataset of the distribution and diversity of these viruses and their related vectors in China (including Taiwan, Hong Kong and Macau). Data was drawn from peer-reviewed journal articles, conference papers and thesis publications in both English and Chinese. Geographical data on mosquito-associated viruses’ occurrence and related mosquito vector species was extracted, and quality-control processes employed. This dataset contains 2,428 accounts of mosquito-associated viruses’ and mosquito species geo-referenced occurrences at various administrative levels in China. The prevalent mosquito-associated virus includes Japanese encephalitis virus, Dengue virus, Banna virus and Culex flavivirus, whereas the abundant mosquito vectors are *Culex tritaeryohynchus*, *Aedes albopictus* and *Culex pipiens pallens*. This geographical dataset delivers a distribution and diversity outline of mosquito-associated viruses in China, and also applicable in various spatial and risk-assessment analysis.

## Abstract (summary)

**Table:** 

Measurement(s)	Geographic Distribution • Diversity • mosquito-borne viruses • Disease Vector
Technology Type(s)	digital curation • longitudinal data analysis
Factor Type(s)	year of data collection
Sample Characteristic - Organism	Culicidae • Viruses
Sample Characteristic - Location	China

Machine-accessible metadata file describing the reported data: 10.6084/m9.figshare.12957806

## Background & Summary

Worldwide, mosquitoes have a vast impact on the global public health. An estimated 3500 species of mosquitoes (family *Culicidae*) are known to exist, of which some are efficient vectors capable of transmitting various human and animal pathogens^1–3^. Some of these mosquito-borne infectious diseases include Zika, Japanese encephalitis, West Nile fever, Dengue fever and Yellow fever. With an increase in incidence and lack of effective prophylaxis and vaccines for some of these mosquito-borne illnesses, significant outbreaks of these diseases levy a substantial burden on global health and economics in various countries^2,4^.

Geographically, China is a vast country that comprises of diverse climatic and ecosystems that are favorable to the propagation of arthropod vectors, more especially the mosquitoes. Lately, China has instituted measures and policies that aim to protect, restore and conserve biodiversity^5–7^, thus it is becoming much more conducive for mosquito persistence. Moreover, the recent rapid increase in trade, domestic eco-tourism and travel within the country has highly presented a potential risk of exposure and exportation of these disease vectors and their associated pathogens to new regions^8^. According to previously published studies, high incidence of mosquito-associated viruses exists in Guangdong, Yunnan, Beijing, Liaoning, Inner Mongolia, Zhejiang and Xinjiang provinces^9–12^. Further, the major mosquito-borne viruses in these high-incidence regions consist of Dengue virus (DENV), Japanese encephalitis virus (JEV), and Tembusu virus (TMUV)^9^. Presently, the literature on mosquitoes and mosquito-associated viruses in China mainly focuses on reporting on ‘region-specific’ findings. However, at present, there exists a gap on a detailed and systematic account of the geographical distribution and diversity of mosquito-associated viruses and their related mosquito vectors in China. There exists a necessity to avail the utmost occurrent information together with their geo-location incidence at finer geographical and administrative levels. Moreover, several early studies reported earlier than 1990 were documented in Chinese, hence our study translates their findings to English, a common language that can be broadly understood and the knowledge be widely disseminated. Wholesomely, this dataset description outlines useful information than can be utilized for future mosquito-borne diseases risk analysis and modelling experiments.

Herein, we describe a dataset of 2,428 published records on geo-referenced distribution and diversity of mosquito-associated viruses and their related mosquito vectors across China, reported between the years 1953 to 2019. The most prevalent mosquito-associated virus being Japanese encephalitis virus, Dengue virus, Banna virus and Culex flavivirus, whereas the most commonly reported mosquito species being *Culex tritaeryohynchus*, *Aedes albopictus* and *Culex pipiens pallens*.

## Methods

### Data collection

**Fig. 1:**
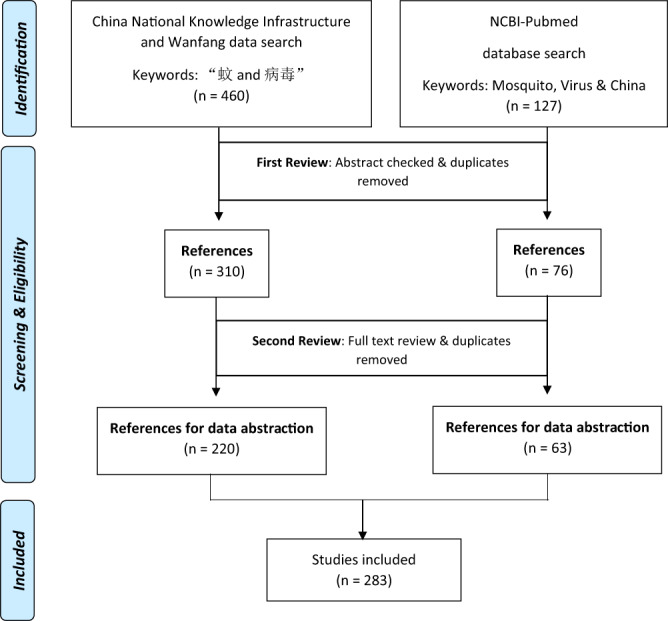
Systematic literature review flow chart of the search strategy and results.

In this overview that spans from January 1953 to December 2019, an intense literature search was conducted on Chinese and English databases. National Center for Biotechnology Information - PubMed was utilized as the central source for English publications while China National Knowledge Infrastructure (CNKI) (http://www.cnki.net/) and Wanfang data (http://www.wanfangdata.com.cn/index.html) were utilized as the source for Chinese publications. The terms (‘Mosquito’, AND ‘Virus’) were used in NCBI PubMed search, and “蚊” for mosquito and “病毒” for the virus were used in the CNKI database. Relevant journal articles, thesis and scientific conference proceedings were retrieved and included in the primary literature search collection. No language limitation was applied. Schematic outline of the literature search is as outlined in Fig. 1.

A total of 587 published manuscripts were retrieved for initial screening (460 Chinese abstracts and 127 English abstracts). Abstracts that solely reported on mosquito species classification and taxonomy were excluded. From this, 310 Chinese and 76 English manuscripts were chosen for a further full-text review. Finally, a total of 283 publications (220 Chinese and 63 English publications) were ascertained as being eligible for extraction. Earliest publications were published as from 1957 to 1990. A detailed list of all the publications that were reviewed and included in this study are presented in the online dataset^13^.

The most significant data extracted from the obtained literature included: (i) Virus name, (ii) GenBank accession number, (iii) Sampling site, (iv) Sampling time, (v) Associated mosquito vector, (vi) Global Positioning System coordinates, and (vii) detection methods (For example PCR, NGS or Cell culture). All the extrapolated data was entered into an excel spreadsheet for downstream analysis. Immediately after, a team of three individuals thoroughly and independently examined the dataset so as to avert possible errors and duplications. In total, 2,428 records of mosquito-associated viruses were gathered from the CNKI, Wanfang data and NCBI PubMed databases.

## Geo-Positioning

Global Positioning System (GPS) coordinates for all the selected studies were extracted from their respective publications. For the manuscripts that only listed their study sites, but no GPS coordinates, we determined the longitude and latitude through coalescing several geospatial tools that include xGeocoding (http://www.gpsspg.com/xgeocoding/), with APIs to access georeferenced functions of the frequently used online maps in China (Baidu Map, Tencent’s QQ Map and Amaps), Google Earth (http://www.google.co.uk/intl/enuk/earth), or as a simple keyword search on Google or Baidu. Where necessary, historical study site names were updated to match the modern administrative names. Further, study site location was categorized into four different levels as per their geographical tiers and administrative levels (i.e. provincial, prefectural, county, and township). We aimed to extract the four-level geographical information for the site where the data was available. In cases where the information was missing, we just left it blank in the dataset. This classification is vital for consumers of this data to excerpt relevant segments for their usage. The distribution of mosquito associated viruses and mosquito species were visualized via open source tools: R v3.5.1 (https://www.r-project.org/), Echarts v4.7.0 (https://echarts.apache.org/zh/index.html) and Openlayer v4.6.5 (https://openlayers.org/). The data map of China with climate zone information were kindly provided by Prof. Tao Pei at Institute of Geographic Sciences and Natural Resources Research, CAS.

## Data Records

In this distribution and diversity dataset of mosquito-associated viruses and their related mosquitoes in China, as accessible from figshare^13^, each dataset row describes a distinct record (incidence of mosquito-associated viruses and related mosquito vectors in a specific location as described at a set time-point in scientific literature). The dataset column details are as follows:

1. ***Category:*** Categorizes whether the identified virus is mosquito-borne or mosquito specific virus
2. ***Virus_name:*** Describes the name of the mosquito-associated virus
3. ***Virus_abbr:*** Describes the abbreviation of the virus
4. ***Virus_strain:*** Describes the strain of the respective mosquito-associated virus
5. ***Virus_genotype:*** Describes the genotype of the respective mosquito-associated virus
6. ***Virus_Genbank No:*** Describes the GenBank number of the respective mosquito-associated virus
7. ***Virus_family:*** Identifies the family-level taxonomy of the mosquito-associated virus
8. ***Virus_genus:*** Identifies the genus-level taxonomy of the mosquito-associated virus
9. ***Mosq_genus:*** Describes the genus of the respective mosquito where the virus was identified.
10. ***Mosq_species:*** Describes the species of the respective mosquito where the virus was identified.
11. **Isolation_status:** Describes the *in vitro* isolation status of the virus through cell culture.
12. ***Nucleic_test_virus:*** Describes if any nucleic acid detection was conducted on the virus, eg PCR assay.
13. ***Sero_test_virus:*** Describes if any serological detection was conducted on the virus
14. ***NGS_test_virus:*** Describes if metagenomic sequencing was used to identity the virus.
15. ***Province_name:*** provincial level, based on China administrative map.
16. ***City_name:*** prefectural level, based on China administrative map.
17. ***County_name:*** county level, based on China administrative map.
18. ***Site_name:*** Describes the township or finer level, based on China administrative map.
19. ***GPS_source:*** details where geographic information (GPS data) was obtained from ‘main manuscript’ or ‘manual geoposition’.
20. ***Long:*** The longitudinal coordinate of the location of mosquito associated virus occurrence. The reference system used is the decimal degrees.
21. ***Lat:*** The latitudinal coordinate of the location of mosquito associated virus occurrence. The reference system used is the decimal degrees.
22. ***smp_start:*** Commencement year of study sampling.
23. ***smp_end:*** Completion year of study sampling.
24. ***pub_year:*** Study publication year.
25. ***Ref_no:*** Describes the reference catalogue number in the list of references (under the list of References in sheet number 2)

## Technical Validation

Herein, this dataset contains 2,428 records that were extracted from 283 literatures that were published between the years 1957 and 2019. At the initial stage, the records were extracted by a team of four members (two members each for English and Chinese publications). Thereafter, one team member compiled the data, cross-checked and confirmed all the entries. At the geo-positioning step, an independent third-party was engaged to re-check the data again. In all the data entry and verification steps, strict quality assessment was done, following a previously described approach by Zhang *et al*.^14^.

**Fig. 2:**
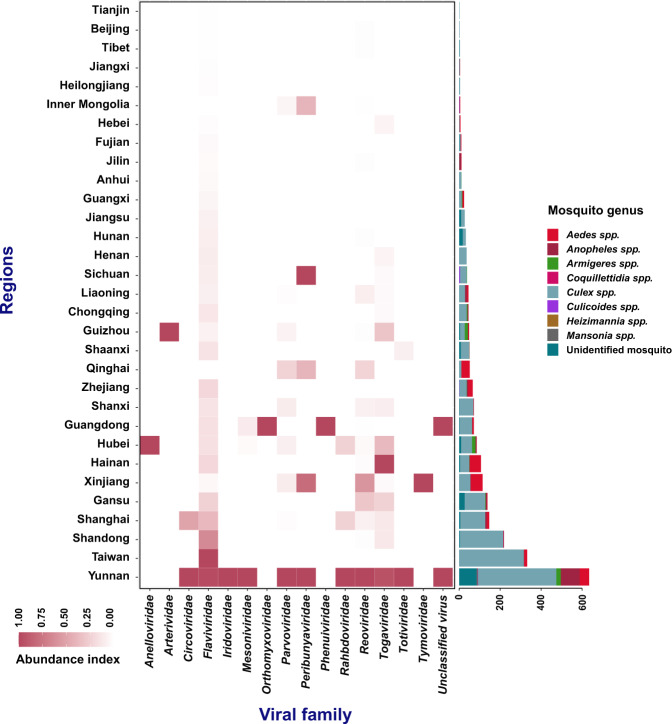
The distribution and diversity of mosquito-associated viruses and the related mosquito genus across the various regions in China. The heatmap indicates the viral family abundance in different regions while the barplot indicates the mosquito genus in the different regions.

**Fig. 3:**
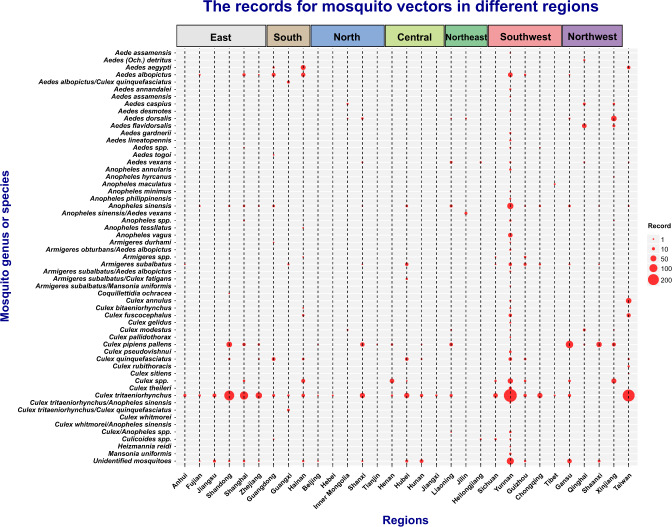
Records of different mosquito vector species across various regions in China. The size of red dot indicates the number of records.

**Fig. 4:**
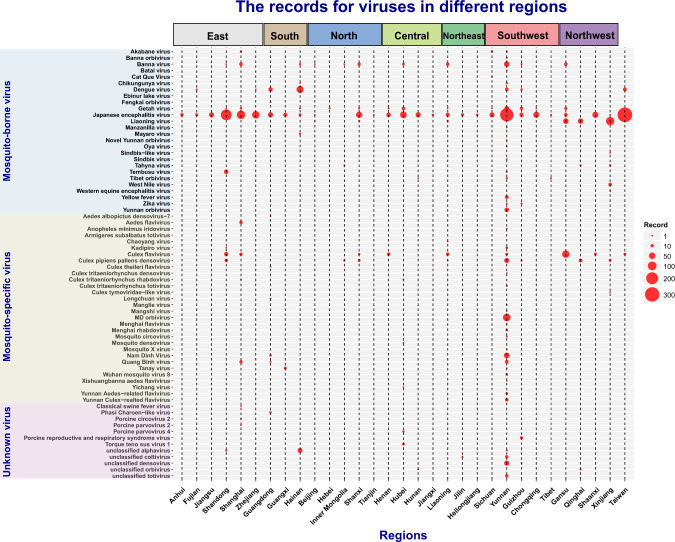
Records of different mosquito associated viruses across various regions in China. The size of red dot indicates the number of records.

**Fig. 5:**
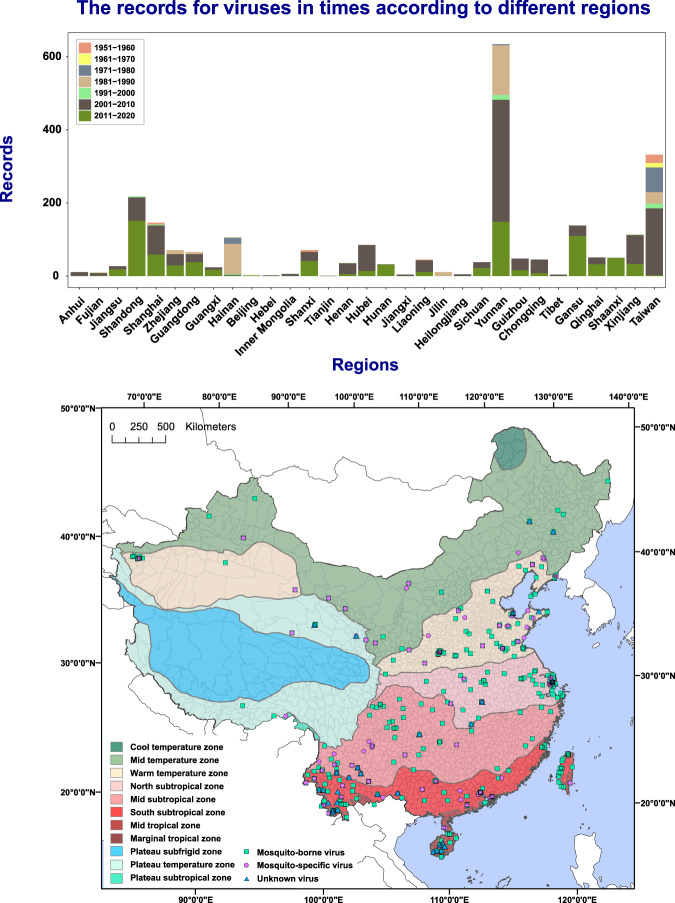
Distribution and density of mosquito-associated viruses by time and climatic zones in China. (**a**) The distribution density of mosquito- associated viruses in different provincial-level divisions of China in different time periods. (**b**) Geo-location distribution of three classes of mosquito-associated viruses based on the climatic regions of China.

It is vital to verify that all locations of mosquito species and the mosquito-associated virus occurrences were appropriately geo-referenced. In some few instances, study sampling sites were incompletely defined, hence it was tough for them to be geo-positioned via the utilized geospatial softwares. For instance, a few study sites were described in their short abbreviation names or local indigenous languages. In other instances, some publications listed the study site names at the very lowest administrative level names in rural parts of China (e.g. names of nearby geographical attraction sites) which could not be readily recognized in any online search sites. To correct these occurrences, our study team conducted a rigorous analysis of all the primary articles while at the same time doing frequent checks on Baidu, Google and analyzing the semantics attained from various sources. Lastly, coordinates mined through xGeocoding were mapped via Google Earth to confirm that each site pointed to the accurate administrative region within China. Notably though, it was not possible for us to get all the variables for complete data entry. This was observed more specifically on dataset entries like virus strain, virus genotype, virus GenBank number, county name, and sample start and end of sampling duration, all of which were left blank. The resultant locations of mosquito-associated viruses’ occurrence and their related mosquito vectors were depicted as illustrated in Figs. 2 to 5.

## Usage Notes

Knowledge of the abundance and diversity of disease vectors is crucial in supporting the making of policies and direct the necessary actions in preventing and management of relevant diseases. Mosquitoes are significant transmitters of arboviruses that are of great global health concern. This dataset serves as the foremost comprehensive compilation of the distribution of mosquito species and mosquito-associated viruses in China (including Taiwan, Hong Kong and Macau). This comprehensive dataset can be applied in Spatio-temporal dynamic investigations of mosquitoes and mosquito-associated virus distribution at multiple geographical scales in China. Additionally, it can as well be used in modelling the possible ecological risks associated with mosquito-borne diseases.

## Data Availability

No custom code was made for the compilation and validation procedures in this dataset.
