# Author Correction: A dataset of distribution and diversity of mosquito-associated viruses and their mosquito vectors in China

**DOI:** 10.1038/s41597-020-00730-9

**Published:** 2020-11-04

**Authors:** Evans Atoni, Lu Zhao, Cheng Hu, Nanjie Ren, Xiaoyu Wang, Mengying Liang, Caroline Mwaliko, Zhiming Yuan, Han Xia

**Affiliations:** 1 Key Laboratory of Special Pathogens and Biosafety, Wuhan Institute of Virology, Chinese Academy of Sciences, Wuhan, Hubei China; 2 University of Chinese Academy of Sciences, Beijing, China; 3 BeiDou SuperSIT Spatial Information Industry (Wuhan) Inc, Wuhan, China

Correction to: *Scientific Data* 10.1038/s41597-020-00687-9, published online 13 October 2020

The Figure 5 has been updated to include the missing part of the “Plateau subtropical zone”, and the subfigure labels, “(a)” and “(b)”.

